# Hydrogen Peroxide in Dental Operations

**Published:** 1892-03

**Authors:** D. D. Peabody

**Affiliations:** Stoneham, Mass.


### HYDROGEN PEROXIDE IN DENTAL OPERATIONS.[1]

BY DR. D. D. PEABODY, STONEHAM, MASS.

Mr. President and Gentlemen,—It was with much diffidence that I yielded to the call of your committee to prepare a paper on any subject for your consideration, because of the feeling that many another could better employ your time; and I consented, only hoping that some suggestion that I may offer may help to lighten the labor and augment the success of some one listening.

Perhaps no problem at present more absorbs the attention of those who have to deal with pathologic conditions than the securing of complete asepsis, and the desirability of accomplishing this result without the complications arising from the use of irritating and, frequently, toxic agents. The last quarter of a century has witnessed no greater advance in medical science than along this line. Those familiar with the scenes in hospitals during the late war recall with a shudder the many brave lives that went out through gangrene and kindred conditions that refused to yield to

---

[1] Read before the New England Dental Society, October 29 and 30, 1891.

the then known and accepted treatment. To-day hospital gangrene is almost unknown, and the similar sloughings and ulcerations have disappeared under the beneficent influence of the antiseptic treatments in common use. As a dental student I was led to believe that most of the septic conditions we are called to face could best be controlled by the use of carbolic acid, creosote, or mercuric chloride; and we can all recall with regret the persistent abscess that ought to have healed, and the putrescent roots that ought to have become sweet and healthy, under their application, and they *would not.* Now, you probably expect me to bring out a hobby, but I am not aware that I have one. I am glad, however, to speak at this time of the satisfaction to be derived from the use of hydrogen peroxide ($H_2O_2$) in many of the various lesions we are called upon to treat in dental surgery. To be effective this agent should contain at least twelve volumes of oxygen to one of water; and as the oxygen escapes or separates from the water as freely as the carbon dioxide from the bottle of ginger ale, it must be kept closely stoppered and not allowed to remain at a temperature above 65° or 70° F. It is better to procure it in four-ounce or eight-ounce cork-stoppered bottles, for ground glass will blow out, and even the corks must be tied in, and should be renewed by fresh ones occasionally, for the efficiency of the oxygen lies in the fact that it is a destroyer. Keep such a bottle in a refrigerator or cool cellar, and use from a small bottle at the case. It is better to shake the large bottle forcibly before opening, to recombine the free oxygen. In view of the great affinity of the oxygen for metals, it should be applied only by means of glass, vulcanite, gold, or platinum, and should never be introduced into a tooth by means of steel or silver instruments. It would seem almost superfluous for me to attempt to instruct this body as to the occasions when the use of hydrogen peroxide is indicated had I not learned by recent inquiry that many dentists have never even seen it in use. When prepared with not more than one per cent. of hydrochloric acid it is non-irritant on all surfaces except the eye, and elsewhere on healthy tissues produces no apparent effect. The writer applied it in the eye of a medical friend a few days since to neutralize the poison received from a diphtheritic patient, without further injury than slight temporary pain, and with complete success, and that in a case where most known antiseptics would be inadmissible.

There is one class of cases in dental practice where its use is contraindicated, because it is *too effective.* I allude to deep-seated abscesses to which ingress is to be obtained only through a small orifice or fistulous opening. Its application to blood or pus is instantly followed by abundant effervescence, which so distends the soft tissues as to effectually close the orifice or outlet, and the pain produced is intolerable. As an instance, a few months since the writer put a few drops into a pustule the size of a pea, near the edge of the gum, the remains of a former abscess, and in a few seconds the patient's eyes were suffused with tears. An examination showed a swelling the size of half a nutmeg, which collapsed at the thrust of a lancet. A medical friend in Somerville last winter injected one-half drachm into a deep perineal abscess following parturition, and was compelled to etherize his patient to relieve the pain and incise for an outlet. Allow me to say, however, that in each of these instances one application effected a cure; but that is not the proper method to use the remedy. For the same reason it should not be forced through the apex of a root of a tooth, unless there is a certainty of a free exit. It is invaluable for injection into the alveolar sockets after extraction of diseased roots, and the writer has not seen a single case of the long-continued soreness and inflammation following extraction which was altogether too frequent previous to its employment. Its effect in the pus-pockets so often found in pyorrhœa alveolaris has been very satisfactory. If present at the operating case, the occasions where its use will be suggested will be innumerable; but I can hardly close this somewhat discursive paper without allusion to its value as a bleaching agent. Fill the apex of a root with gutta-percha, and the canal and pulp-chamber with freshly-dried aluminum chloride, and saturate with the peroxide. Allow it to remain five or ten minutes, and wash out with sodium bicarbonate. Credit for this formula is due to Dr. A. W. Harlan, of Chicago, and I have yet to find a more satisfactory method.

